# Short hydrophobic loop motifs in BRICHOS domains determine chaperone activity against amorphous protein aggregation but not against amyloid formation

**DOI:** 10.1038/s42003-023-04883-2

**Published:** 2023-05-08

**Authors:** Gefei Chen, Axel Leppert, Helen Poska, Harriet E. Nilsson, Carlos Piedrafita Alvira, Xueying Zhong, Philip Koeck, Caroline Jegerschöld, Axel Abelein, Hans Hebert, Jan Johansson

**Affiliations:** 1 Department of Biosciences and Nutrition, Karolinska Institutet, 141 83 Huddinge, Sweden; 2 School of Natural Sciences and Health, Tallinn University, Tallinn, Estonia; 3 School of Engineering Sciences in Chemistry, Biotechnology and Health, Department of Biomedical Engineering and Health Systems, KTH Royal Institute of Technology, 141 52 Huddinge, Sweden; 4 Present Address: Department of Microbiology, Tumour and Cell Biology, Karolinska Institutet, 171 65 Solna, Sweden

**Keywords:** Biochemistry, Proteins

## Abstract

ATP-independent molecular chaperones are important for maintaining cellular fitness but the molecular determinants for preventing aggregation of partly unfolded protein substrates remain unclear, particularly regarding assembly state and basis for substrate recognition. The BRICHOS domain can perform small heat shock (sHSP)-like chaperone functions to widely different degrees depending on its assembly state and sequence. Here, we observed three hydrophobic sequence motifs in chaperone-active domains, and found that they get surface-exposed when the BRICHOS domain assembles into larger oligomers. Studies of loop-swap variants and site-specific mutants further revealed that the biological hydrophobicities of the three short motifs linearly correlate with the efficiency to prevent amorphous protein aggregation. At the same time, they do not at all correlate with the ability to prevent ordered amyloid fibril formation. The linear correlations also accurately predict activities of chimeras containing short hydrophobic sequence motifs from a sHSP that is unrelated to BRICHOS. Our data indicate that short, exposed hydrophobic motifs brought together by oligomerisation are sufficient and necessary for efficient chaperone activity against amorphous protein aggregation.

## Abstract (web summary)

BRICHOS domain oligomerization exposes three short hydrophobic motifs that are necessary for efficient chaperone activity against amorphous protein aggregation.

## Introduction

Molecular chaperones are central to the proteostasis machinery by assisting correct folding of polypeptides^1,2^. Among them, ATP-independent molecular chaperones, like the small heat shock proteins (sHSPs) prevent toxic consequences of protein aggregation by binding to un- or misfolded substrates and keeping them in a soluble, refolding competent state^3,4^. Bri2 is a type II transmembrane protein, ubiquitously expressed, and its proteolytic processing releases a molecular chaperone domain—BRICHOS^5,6^. Interestingly, other molecular chaperones like HSP60, HSP70, and HSP90 are part of the Bri2 interactome in the brain and retina^7,8^. The isolated Bri2 BRICHOS domain modulates the aggregation pathways of several amyloid-forming substrates, averts amyloid-associated toxicity, and prevents non-fibrillar, amorphous protein aggregation, similar to ATP-independent molecular chaperones like crystallins or clusterin^9–13^. The ability of the Bri2 BRICHOS domain to interact with substrates involved in protein aggregation disorders like Alzheimer’s disease^14–16^, type II diabetes^17^, and Parkinson’s disease^18^ highlights its potential importance for cellular proteostasis.

**Fig. 1:**
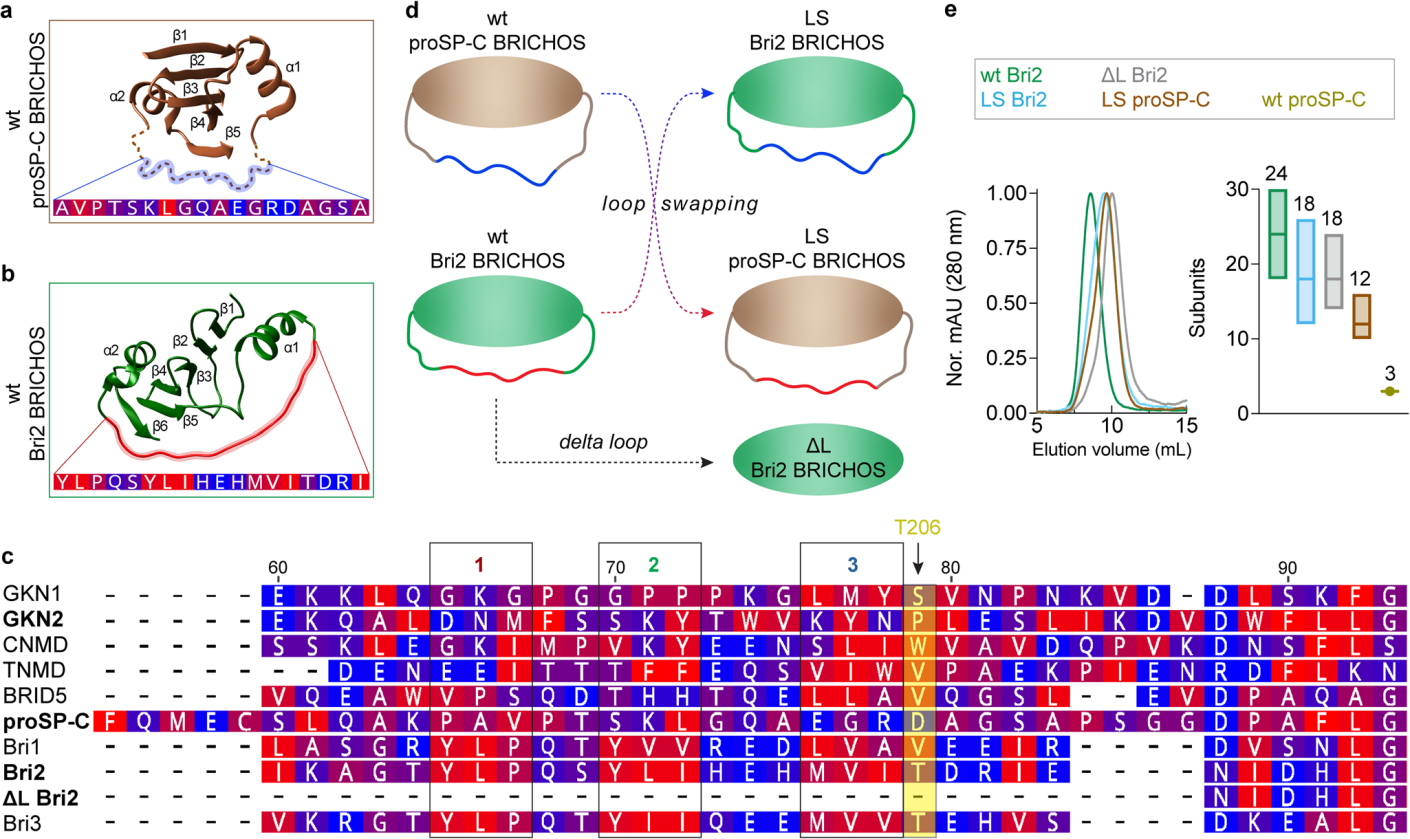
BRICHOS loop regions and loop-swap variants. **a** Single subunit from the crystal structure of wt proSP-C BRICHOS trimers (PDB accession number: 2yad). The dotted line indicates the loop which is missing in the crystal structure and its core region is marked with a blue shadow. The sequence is labeled in increments from blue for polar to red for hydrophobic residues. **b** Wt Bri2 BRICHOS modeled by AlphaFold 2, where the loop core is labeled as in (**a**). **c** Amino acid sequences of human BRICHOS loop regions (see Supplementary Fig. 1 for alignment) color-coded as in (**a**, **b**). The BRICHOS domains studied herein are in bold, the motifs 1–3 are boxed and T206 is indicated with an arrow. **d** Schematic models of loop swap and delta loop variants. **e** SEC of BRICHOS oligomer variants (left) and estimated number of subunits (right). Number of subunits were estimated from the SEC peak maxima (indicated by center lines and numbers above boxes) and the boxes represent their full width at half maximum.

The BRICHOS domain is about 100 amino acid residues in size and exists as monomers or multimers ranging from dimers to large polydisperse oligomers stabilized by covalent and non-covalent interactions^11,19,20^. The human Bri2, and Bri3, BRICHOS domains assemble into large oligomers of about 20–30 subunits, which efficiently inhibit amorphous protein aggregation. In contrast, Bri2 BRICHOS monomers and dimers, as well as the BRICHOS domain from prosurfactant protein C (proSP-C), which mainly exists as trimers, are all inactive against amorphous protein aggregation^9,11,19,20^. The BRICHOS domains from different families have low pairwise sequence identities but homology models of the Bri2 BRICHOS monomer based on the proSP-C BRICHOS crystal structure show a conserved central five-stranded β-sheet that is flanked by two α-helices connected via a long flexible loop (Fig. 1a, b)^19–22^. To date, there is no experimental structure to atomic detail available for the Bri2 BRICHOS domain, probably related to the facts that the monomeric subunit is conformationally dynamic^23^, and that the larger oligomers are polydisperse (see further below).

Classical ATP-independent sHSP molecular chaperones, like αB-crystallin, assemble into large polydisperse and dynamic complexes and can bind to a diverse set of protein substrates^3,10^. Data suggest that the structural plasticity of ATP-independent molecular chaperones mediates the exposure of individual binding regions for either amyloid or amorphous clients. While the binding sites for amyloid substrates might depend on the substrate and type of fibrillar aggregate^24,25^, the flexible and partially disordered N-terminal domains (NTDs) of sHSPs, enriched in hydrophobic residues, appear important for the formation of high-molecular-weight assemblies and interaction with amorphously aggregating model substrates^25–29^.

In this study, we aimed to understand the architecture of the Bri2 BRICHOS domain oligomers, and define the client binding site regarding the chaperone activity against amorphous protein aggregation. Our results show that three short hydrophobic motifs in an unstructured loop of the Bri2 BRICHOS domain constitute the key region for stabilizing partly unfolded substrates against amorphous protein aggregation, are important for oligomer plasticity but do not dictate the ability to retard amyloid formation.

## Results

### Bri2 BRICHOS loop is important for oligomerization and inhibition of amorphous protein aggregation

In search for an explanation of the distinct behaviors of human BRICHOS proteins in terms of activity to prevent amorphous protein aggregation, we recognized that the loop connecting α-helices 1 and 2 contains three hydrophobic tripeptide motifs in the chaperone-active Bri2 and Bri3 BRICHOS, whereas the corresponding motifs in the chaperone-inactive proSP-C BRICHOS are polar and charged (Fig. 1a–c, Supplementary Fig. 1). To test whether the loop defines the ability to form oligomers and to prevent amorphous protein aggregation, we swapped the loops between human Bri2 and proSP-C BRICHOS, thus generating LS (loop swap) variants (Fig. 1d). Bri2 BRICHOS from *Ictidomys tridecemlineatus* (NCBI accession number: XP_013216182) and *Octodon degus* (NCBI accession number: XP_004633182) lack the entire loop but are otherwise almost identical to human Bri2 BRICHOS and are here referred to as delta loop (ΔL) Bri2 BRICHOS (Fig. 1c, d, Supplementary Fig. 1). Recombinant LS and ΔL Bri2 BRICHOS exhibit similar oligomerization profiles on size exclusion chromatography (SEC) and overall secondary structures of isolated oligomers, dimers and monomers as the wild type (wt) human Bri2 BRICHOS counterparts^11^ (Supplementary Fig. 2a–f). LS proSP-C BRICHOS forms large polydisperse disulfide-linked oligomers, in addition to trimers and monomers that are observed for wt proSP-C BRICHOS^19^ (Supplementary Fig. 2g–i). LS Bri2, LS proSP-C and ΔL Bri2 BRICHOS oligomers have overall fewer subunits (about 12–24) than wt Bri2 BRICHOS oligomers (about 18–30) (Fig. 1e).

**Fig. 2:**
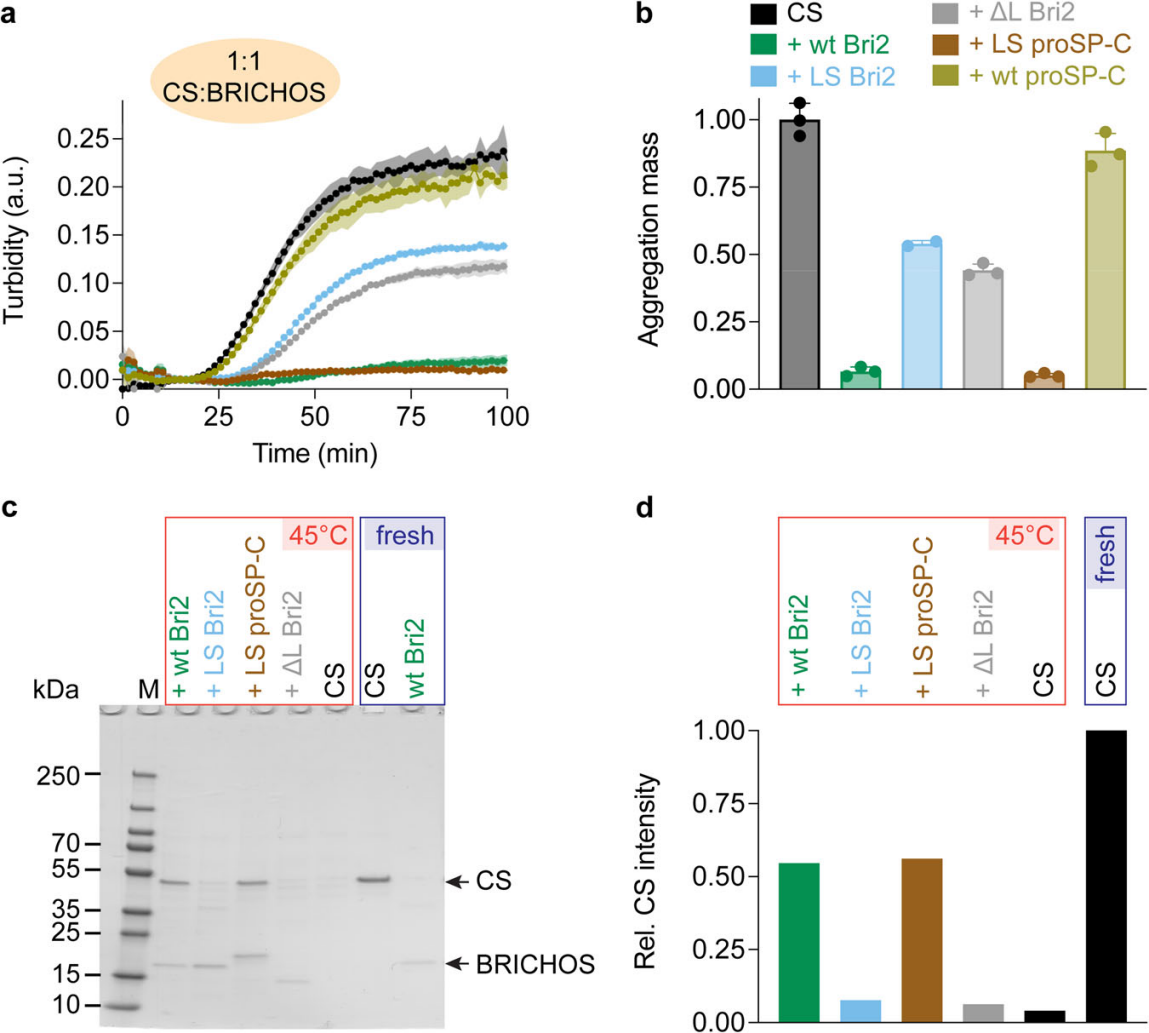
Ability of BRICHOS loop variants to suppress amorphous protein aggregation. **a** Aggregation traces of 1.2 µmol L^-1^ CS at 45 °C alone and with the different loop variants at molar ratio of 1:1 (CS: BRICHOS), color-coded as in (**b**). The aggregation traces are from 2–3 replicates. **b** Aggregation mass determined from the areas under the curves in (**a**). Data are presented as means ± standard deviations and normalized to the aggregation mass of CS only. **c** Mixtures of CS with and without BRICHOS loop variants after incubation at 45 °C, or non-incubated fresh samples, analyzed by SDS-PAGE. **d** Band intensities in (**c**) assessed by ImageJ^50^ and normalized to the band intensity of fresh CS.

Next, we followed the aggregation kinetics of the model substrates citrate synthase (CS) and rhodanese (Rho), which unfold and form amorphous aggregates at elevated temperatures, in the absence and the presence of oligomeric fractions of all BRICHOS loop variants. LS Bri2 and ΔL Bri2 BRICHOS have significantly reduced activities against both CS (Fig. 2a, b, Supplementary Fig. 3a, b) and Rho (Supplementary Fig. 3c, d) compared to wt Bri2 BRICHOS. In contrast, LS proSP-C BRICHOS is equally efficient as wt Bri2 BRICHOS in preventing amorphous protein aggregation, while wt proSP-C BRICHOS trimers lack chaperone activity (Fig. 2a, b, Supplementary Fig. 3a–d)^9^. Analysis of soluble and insoluble fractions of CS after the aggregation had reached a plateau corroborated that proteins containing the hydrophobic Bri2 BRICHOS loop are more potent in maintaining the substrate in a soluble state (Fig. 2c, d). LS Bri2 and ΔL Bri2 BRICHOS have some residual activity in the turbidity aggregation assays, in particular at equimolar ratios between CS and the BRICHOS proteins (Fig. 2a, b, Supplementary Fig. 3a–d) but they are completely unable to keep CS in solution (Fig. 2c, d). The reason for this discrepancy remains to be determined but LS Bri2 and ΔL Bri2 BRICHOS might affect the sizes of CS and Rho aggregates and thereby reduce their ability to scatter light. To further investigate the effects of the Bri2 BRICHOS loop on the ability to prevent non-fibrillar aggregation, a fusion protein containing the chaperone-inactive solubility tag NT*^30^ and the isolated loop (NT*-loop) were produced. Strikingly, NT*-loop oligomers (about 20 subunits, estimated by SEC) are able to reduce CS aggregation by about 40%, while NT*-loop monomers are inactive (Supplementary Fig. 3e). Even though the chaperone efficiency of NT*-loop oligomers is lower than wt Bri2 BRICHOS oligomers, the results still suggest that bringing multiple Bri2 BRICHOS loops into proximity on NT*-loop oligomers collectively promotes chaperone activity.

To evaluate whether the activity against amyloid formation is affected by the presence of the loop, thioflavin T (ThT) was used to trace the kinetics of Aβ42 fibril formation^31^. ΔL Bri2 BRICHOS, independent of its assembly state, has similar efficiency against Aβ42 fibril formation as wt Bri2 BRICHOS monomers (Supplementary Fig. 4a, b). Global kinetic fitting of microscopic rate constants^32–34^ showed that secondary nucleation and elongation during Aβ42 fibril formation are affected in a similar manner by ΔL Bri2 BRICHOS as demonstrated for wt Bri2 BRICHOS (Supplementary Fig. 4c–e)^11,15^. Furthermore, isolated Bri2 BRICHOS loop monomers, NT*-loop monomers or oligomers were tested against Aβ42 fibril formation at 37 °C, a temperature at which the isolated loop is unstructured and stable (Supplementary Fig. 3f). The isolated loop or NT*-loop monomers did not show any ability to suppress Aβ42 fibril formation (Supplementary Fig. 3g). NT*-loop oligomers slightly delayed Aβ42 fibril formation, but the effect was not concentration dependent (Supplementary Fig. 3h).

### Bri2 BRICHOS loop is solvent-exposed and involved in oligomerization

**Fig. 3:**
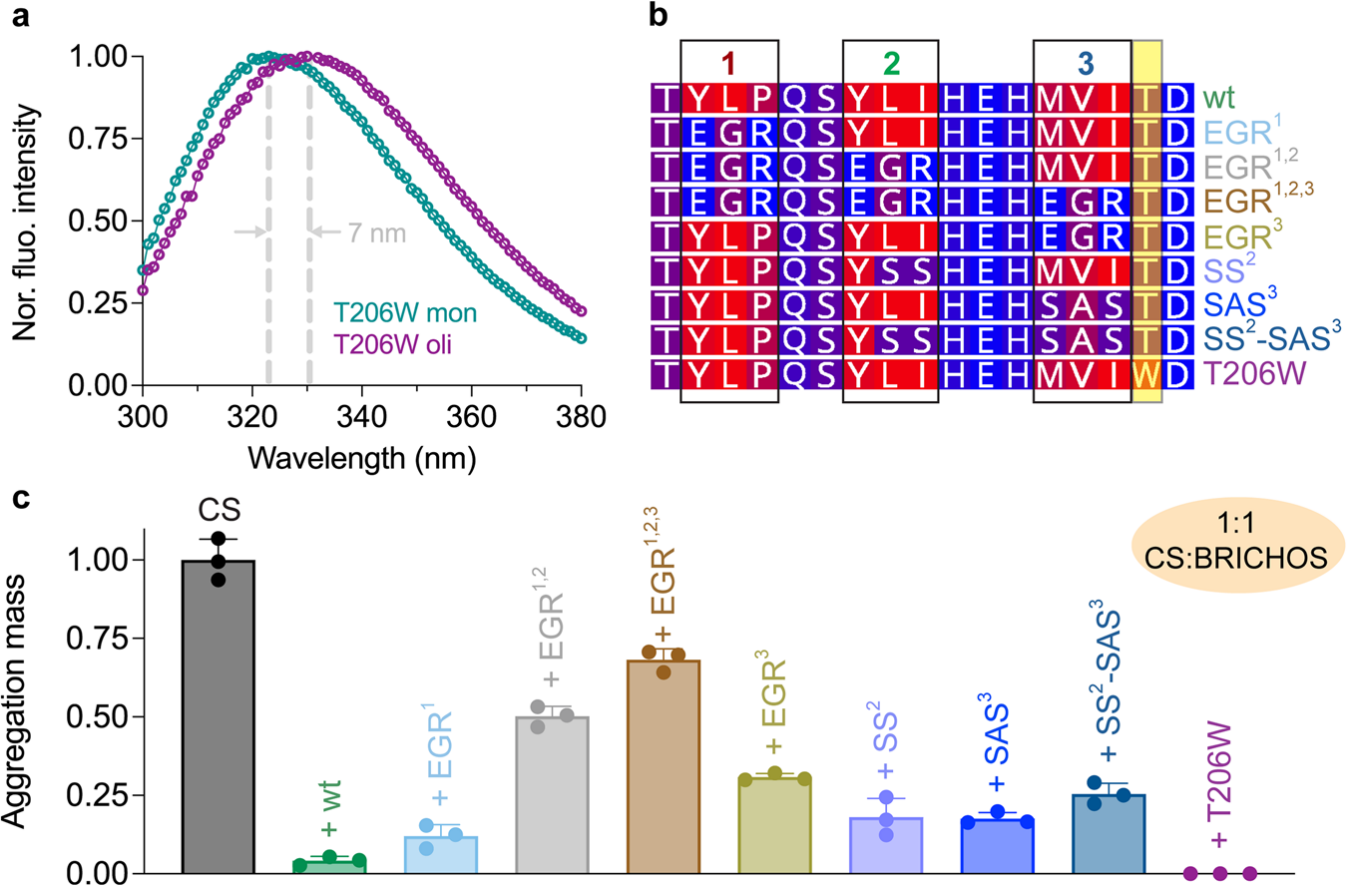
Bis-ANS binding and the importance of hydrophobic motifs for chaperone activity of Bri2 BRICHOS. **a** Normalized Trp fluorescence for rh Bri2 BRICHOS T206W monomers and oligomers. **b** Amino acid sequences of loop motifs 1–3 mutants (boxed), see Fig. 1 for entire loop sequences. **c** Aggregation mass of 1.2 µmol L^−1^ CS at 45 °C in the absence or presence of the different loop mutants at 1:1 (CS: BRICHOS) molar ratio. The aggregation mass are from three replicates. Data are presented as means ± standard deviations.

Bri2 BRICHOS oligomers incubated together with CS at elevated temperature form a complex that can be isolated by SEC (Supplementary Fig. 5a). SDS-PAGE of the complex isolated by SEC showed two bands, corresponding to CS and rh Bri2 BRICHOS, respectively, while native PAGE only revealed one band (Supplementary Fig. 5b). Negative-stain EM of the Bri2 BRICHOS oligomers and CS complex (Supplementary Fig. 5c) show particles that are structurally different from those seen by negative-stain EM of Bri2 BRICHOS oligomers alone^11^. These results suggest Bri2 BRICHOS oligomers can prevent amorphous protein aggregation by forming complex with partly denatured substrates. To probe the solvent accessibility of the Bri2 BRICHOS loop in active oligomers, we designed a reporter system based on Trp fluorescence (Fig. 3a). Since Bri2 BRICHOS does not contain any Trp residues we replaced Thr at position 206, located immediately after the third hydrophobic motif in the loop, with Trp (T206W) (Fig. 3b). When excited at 280 nm rh Bri2 BRICHOS T206W oligomers exhibit a Trp fluorescence emission maximum at 330 nm while the monomers present a emission maximum at 323 nm (Fig. 3a), indicating that the microenvironment of the Trp206 becomes more polar during BRICHOS monomer to oligomer transformation. These findings encouraged us to investigate in more detail to what extent the hydrophobic motifs in the Bri2 BRICHOS loop are crucial to its chaperone function.

### Hydrophobicity of short motifs correlates with chaperone efficiency against amorphous aggregation but not against amyloid formation

We created a set of Bri2 BRICHOS mutants with the aim to modulate the hydrophobicity and charge of the three tripeptide motifs in the loop (Fig. 3b). In four mutants the residues in different tripeptide motifs were mutated to glutamic acid-glycine-arginine (EGR) (Fig. 3b), as in motif 3 of chaperone-inactive wt proSP-C BRICHOS (Fig. 1c, Supplementary Fig. 1). Three further mutants have replacements of hydrophobic residues with the more polar residues serine and alanine (SS and SAS), and the T206W in contrast is more hydrophobic than the wt protein (Fig. 3b). All mutants formed oligomers with similar secondary structures as wt Bri2 BRICHOS but the average sizes of all mutant oligomers were somewhat smaller than the wt oligomers (Supplementary Figs. 6, 7). Mutants that are less hydrophobic than wt Bri2 BRICHOS were also less efficient in inhibiting thermo-induced amorphous aggregation of CS and Rho, while the more hydrophobic T206W mutant showed slightly enhanced efficiency (Fig. 3c, Supplementary Fig. 8a, b).

**Fig. 4:**
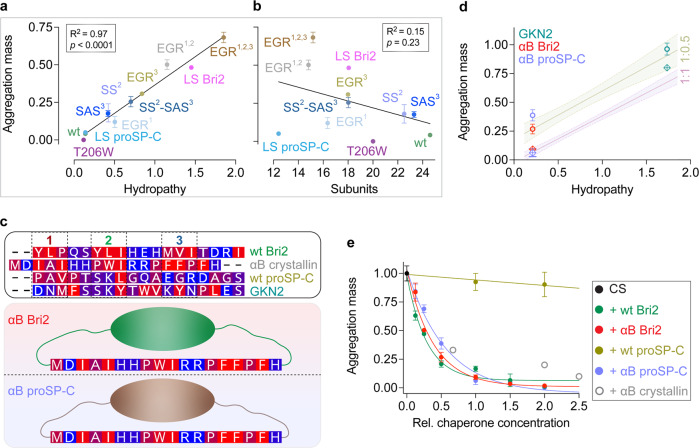
Correlation analysis and predictions for other chaperones. **a**, **b** Correlation analysis between the aggregation mass and hydropathy of motifs 1-3 plus T206 or W206, calculated using the biological hydrophobicity scale^35^ (**a**) and number of subunits in oligomers calculated from SEC data in Supplementary Fig. 7. (**b**). **c** Top panel, amino acid sequences of loops from wt Bri2, proSP-C and GKN2 BRICHOS, and the first 18 residues of the NTD of αB-crystallin (see Supplementary Fig. 11a). Middle and bottom panels, schematic models of αB Bri2 and αB proSP-C BRICHOS chimeras. **d** Linear regressions of relationships between CS aggregation mass and hydropathy derived from BRICHOS variants at 1:1 (data from **a**, left panel) or 1:0.5 (data from Supplementary Fig. 8c) CS:BRICHOS ratios, with 95% confidence band indicated by dashed lines and shadow. Experimentally determined CS aggregation masses in the presence of human GKN2 BRICHOS (green), αB Bri2 (red) or αB proSP-C BRICHOS chimeras (light blue) (rhombuses 1:1 ratios, circles 1:0.5 ratios) plotted with error bars at the hydropathy values calculated for their respective combined motifs 1–3 (see (**b**) for sequences). **e** The aggregation masses of 1.2 µmol L^−1^ CS at 45 °C with and without different concentrations of wt Bri2 and proSP-C BRICHOS domain oligomers, and αB Bri2 and αB proSP-C BRICHOS chimera oligomers. The data points were fitted to exponential or linear decay functions. The data are presented as means ± standard deviations. Activities of recombinant human αB-crystallin from literature values^39–41^ are plotted with gray circles as a reference.

There is a strong and significant linear correlation between the overall, combined hydrophobicity of the three motifs of the loop and the chaperoning efficiency against destabilized CS and Rho (Fig. 4a, Supplementary Fig. 8c, d). Here, hydropathies were calculated using the “biological” hydrophobicity scale, which is derived from the ability of each amino acid residue to be inserted into the endoplasmic reticulum lipid membrane^35^. Using the Kyte-Doolittle scale^36^ to describe the hydropahy of the motifs resulted in qualitatively similar correlations (Supplementary Fig. 9). This suggests that Bri2 BRICHOS oligomers recognize exposed hydrophobic regions in the partly unfolded substrates mainly via three short hydrophobic motifs separated by short polar or charged segments. Since the residue composition of the loop also affects the degree of oligomer assembly (Fig. 1e), we correlated the number of constituent subunits estimated from SEC data (Supplementary Fig. 7a) with chaperone activity as well as with hydrophobicity of loop motifs. For the activities against the substrates CS and Rho, there is no correlation between the number of subunits in oligomers and chaperone activity (Fig. 4b, Supplementary Fig. 8e, f). If the same analysis is restricted to only the variants that contain the Bri2 BRICHOS scaffold, i.e. leaving LS proSP-C BRICHOS out, there is a weak tendency that larger oligomers are more active against CS (*R*^2^ = 0.5, *p* < 0.05), but still no correlation is found for the activity against Rho (*R*^2^ = 0.2, *p* = 0.19). No correlation was detected between the motif hydropathies and the number of constituent subunits of the chaperone oligomers, although all mutant oligomers had somewhat smaller average sizes than the wt Bri2 BRICHOS counterpart (Supplementary Fig. 8g). Thus, while Bri2 BRICHOS monomers, dimers and tetramers are completely inactive against amorphous protein aggregation^11,37^, the exact number of subunits does not determine the activity of larger oligomers (Fig. 4b, Supplementary Fig. 8e, f). In line with this conclusion, it can be noted that the LS proSP-C BRICHOS circa 12-mers are equally efficient as wt Bri2 BRICHOS oligomers which have about twice the number of subunits (Figs. 1e, 2b, d).

All mutants as well as ΔL Bri2 BRICHOS were efficient against Aβ42 fibril formation, although to different degrees (Supplementary Fig. 4 and 10a–h). No correlation was detected between the ability to delay the half-time of amyloid fibril formation (τ_1/2_) and (i) the loop motif hydropathy or (ii) oligomer size (Supplementary Fig. 10i, j). This suggests that while chaperone activity against amorphous protein aggregation of BRICHOS oligomers relies on exposed hydrophobic regions, the targeting of amyloid formation is based on other principles. This conclusion agrees well with the recent finding that αB-crystallin binding to α-synuclein fibrils occurs *via* monomers and is driven by entropy rather than *via* oligomers and hydrophobic interactions^38^.

### Hydrophobicities of short loop motifs determine chaperone activities of BRICHOS oligomers

Next, we asked whether the hydropathies of short loop motifs of other ATP-independent molecular chaperones determine their activities against amorphous protein aggregation. From the linear correlations between the loop motif hydrophobicity and chaperone activity against amorphous aggregation (Fig. 4a, Supplementary Fig. 8c), we predicted, that the BRICHOS domain of human gastrokine 2 (GKN2) (Fig. 1c, Supplementary Fig. 1) would reduce the CS aggregation mass to 65% (95% confidence interval (CI) 61–68%) and 91% (95% CI 84–97%) at 1:1 and 1:0.5 ratio, respectively (Fig. 4d). The corresponding experimental results for recombinant wt GKN2 BRICHOS oligomers were 80 ± 1.2% and 96 ± 5.2%, respectively (Fig. 4d). The predictions from these three loop motifs are surprisingly accurate considering that the overall pairwise sequence identity between the entire Bri2 and GKN2 BRICHOS domains is 20%.

To investigate whether the correlation between loop motif hydrophobicity and chaperone activity extends to sHSPs we turned to αB-crystallin. The NTD of the sHSP αB-crystallin (also HSPB5) is partly disordered, has several hydrophobic motifs and has been shown to be important for binding of amorphous substrates^25^. We replaced part of the loops of wt Bri2 and wt proSP-C BRICHOS with the N-terminal eighteen residues of αB-crystallin. This αB-crystallin NTD segment has a similar hydrophobic motif pattern as the loop of wt Bri2 BRICHOS, but no sequence homology to the loops of either of the BRICHOS domains (Fig. 4c, Supplementary Fig. 11a). The resulting αB Bri2 and αB proSP-C BRICHOS chimeras (Fig. 4c) form large polydisperse oligomers, roughly 12–26 mers and 16–28 mers, respectively, and adopt identical overall secondary structures compared to wt Bri2 BRICHOS oligomers (Supplementary Fig. 11b, c). Oligomers of αB Bri2 and αB proSP-C BRICHOS chimeras are as efficient inhibitors of amorphous protein aggregation as wt human αB-crystallin^39–41^ or wt Bri2 BRICHOS, while wt proSP-C BRICHOS is completely inactive (Fig. 4e). Intriguingly, for both chimeras, the efficiencies against amorphous protein aggregation predicted from only the biological hydrophobicity of the three motifs in the αB-crystallin NTD, using the linear regression derived from our data (Fig. 4a, Supplementary Fig. 8c), fit almost exactly with the corresponding experimental data (Fig. 4d).

## Discussion

The data presented herein show that the ability of the Bri2 BRICHOS domain to prevent partly denatured substrate proteins from forming amorphous aggregates is strongly correlated to hydrophobicity of three short motifs that are located in a loop region and interspersed by short polar stretches. This suggests that direct interactions between exposed hydrophobic regions in the partly denatured substrates and the hydrophobic loop motifs of the chaperone are central for the ability to suppress amorphous aggregation. The fact that monomeric, dimeric or tetrameric Bri2 BRICHOS are completely inactive in preventing amorphous protein aggregation^11,37^ points to that formation of larger oligomers is essential. We found that the hydrophobicity of the three short loop motifs ultimately determines the chaperone activity seemingly by defining the affinity of the substrate-binding site as well as the quaternary structural arrangement in BRICHOS oligomers. The essential importance of the loop hydrophobicity was perhaps most clearly demonstrated by loop-swaps from Bri2 BRICHOS or αB-crystallin, respectively, to proSP-C BRICHOS, which resulted in highly efficient oligomeric chaperones, in contrast to the essentially completely inefficient wt proSP-C BRICHOS trimers (Fig. 4e).

Molecular chaperones are believed to use intrinsically disordered regions for the recognition of a wide variety of misfolding substrates. ATP-independent molecular chaperones like HSP33, HSP21 or HdeA partially unfold under stress conditions to efficiently bind misfolded clients^42–44^. It is intriguing that the hydrophobic motifs here determined to be essential for chaperone activity of Bri2 BRICHOS oligomers are in a supposedly unstructured loop region. The loop configuration and hence exposed binding sites in a Bri2 BRICHOS oligomer is likely highly dynamic allowing to present variable clusters of hydrophobic residues which contribute to the ability to interact with diverse substrates. Furthermore, the NTD within the sHSP family is highly variable in sequence and length but has been demonstrated to be the key to their ability to bind various substrates^25,28,45^. Our results strongly support that exposure of flexible disordered hydrophobic patches rather than sequence identity mediate the substrate plasticity and efficiency against amorphous protein aggregation. The ability to form oligomers and expose hydrophobic motifs is shared between BRICHOS and crystallins but these two families are otherwise structurally different. As shown here, BRICHOS needs to form larger oligomers that bring together and expose hydrophobic motifs to gain efficient canonical chaperone activity, while crystallins generally get more active upon dissociation into smaller subunits^27^. The apparently simple strategy to enable BRICHOS-substrate interactions by bringing together short loop motifs can be a manner to regulate chaperone activity under physiological conditions^46^ and mutagenesis of the BRICHOS domain might be a way to generate novel chaperone functions.

## Methods

### Protein preparation

All variants of the recombinant human Bri2 BRICHOS domain (residues 113 to 231 of human Bri2 BRICHOS), LS Bri2 BRICHOS, ΔL Bri2 BRICHOS, the BRICHOS chimeras αB Bri2 BRICHOS and αB proSP-C BRICHOS that harbor residues 1–18 of human αB crystallin (HspB5, NCBI accession number: P02511), and the isolated Bri2 BRICHOS loop (residues 187 to 216 of human Bri2 protein) were cloned into a modified pET32 vector containing the spider silk derived His6-NT* tag^30^, and different assembly states were purified^11^. Human proSP-C BRICHOS, corresponding to amino acid residues 59–197, was prepared^6^ and oligomers of LS proSP-C BRICHOS were isolated by size exclusion chromatography (SEC) using a Superdex 200 column (GE Healthcare) as an additional last step. The gene fragment of the human GKN2 BRICHOS domain, corresponding to residues 21 to 151 of human GKN2, was cloned into a modified pET32 vector containing the His6-NT* tag and expressed and purified according to the protocol used for Bri2 BRICHOS. Mutations were introduced via PCR using KAPA HiFi HotStart ReadyMix PCR Kit (Kapa Biosystems, USA) or constructs were synthesized by GenScript biotech. All sequences were confirmed by DNA sequencing (Eurofins Genomics). BRICHOS protein concentrations were determined by measuring the absorbance at 280 nm using the respective extinction coefficients. All concentrations given are based on monomeric subunits.

### CD spectroscopy, analytical SEC and Trp fluorescence measurements

Circular dichroism (CD) spectra were recorded at 25 °C in a J-1500 CD spectrophotometer (JASCO) with a PTC-517 Peltier thermostat cell holder. Five or 10 µmol L^−1^ protein samples were prepared in 20 mmol L^−1^ NaPi, 0.2 mmol L^−1^ EDTA, pH 8.0 in a quartz glass cuvette with a 1 mmol L^−1^ path length. Five consecutive scans per sample between 180 and 260 nm, with an increment of 0.5 nm, were acquired. Averaged and blank subtracted spectra were converted to the mean residue ellipticity (MRE, deg cm^2^ dmol^−1^).

Analytical SEC was performed using a Superdex 200 increase column using a 500 µL sample loop. 10 µmol L^−1^ protein samples were prepared in 20 mmol L^−1^ NaPi, 0.2 mmol L^−1^ EDTA, pH 8.0, and the same buffer was used as SEC running buffer.

For tryptophan fluorescence measurements, recombinant proteins were prepared in triplicates in 20 mM NaPi pH 8.0 with 0.2 mM EDTA with a volume of 150 uL in black polystyrene flat-bottom 96-well plates (Costar) and excited at 280 nm (5 mm bandwidth). Fluorescence emission from 300–400 nm (10 mm bandwidth, 1 nm step interval) was recorded using a spectrofluorometer (Tecan Saphire 2). Data were corrected by subtracting the background fluorescence (buffer).

### Evaluation of chaperone activity against amorphous protein aggregation

Thermo-induced aggregation of 0.6 µmol L^−1^ porcine heart citrate synthase (CS) (Sigma-Aldrich, Germany) and 3 µmol L^−1^ bovine liver rhodanese (Rho) (Merck; Darmstadt, Germany) were followed by measuring the absorbance at 360 nm in 90 s cycles during incubation at 45 °C using a POLARstar Omega plate reader (BMG Labtech, Offenberg, Germany). Samples were prepared in black, clear-bottom half-area polystyrene 96-well plates (Corning Glass 3881, USA) and the reactant volume was 100 µL. Stock solutions of the model substrates were prepared in either 40 mmol L^−1^ HEPES/KOH, pH 7.5 (for CS), or PBS, pH 7.4 (for Rho) and diluted in buffer containing 40 mmol L^−1^ HEPES/KOH, pH 7.5 (for CS) or PBS, pH 7.4 (for Rho). Samples were complemented with 20 mmol L^−1^ NaPi, 0.2 mmol L^−1^ EDTA, pH 8.0 for equal buffering conditions from BRICHOS proteins. Measurements were performed in triplicates and aggregation kinetics analyzed by integrating the area under the curve, expressed as aggregation mass normalized to the aggregation of CS alone. Molar ratios indicated refer to monomers. For complex formation, 0.6 µmol L^−1^ CS (Sigma-Aldrich, Germany) was incubated with 6 µmol L^−1^ rh Bri2 BRICHOS oligomers in 40 mmol L^−1^ HEPES/KOH pH 7.5 at 45 °C using a POLARstar Omega plate reader (BMG Labtech, Offenberg, Germany). The final product was injected into a superose 6 column (Cytiva) and eluted with 20 mmol L^−1^ NaPi pH 8.0 containing 0.2 mmol L^−1^ EDTA, and different fractions were collected for SDS-PAGE and native PAGE analysis. The BRICHOS-CS complex was applied to carbon-coated copper grids (400 mesh, Analytical Standards), negative-stained with 1% phosphotungstic acid (PTA) and observed under transmission electron microscopy (TEM, Jeol JEM2100F at 200 kV).

### Aβ42 monomer preparation and ThT assay

Recombinant Aβ(1–42), here referred to as Aβ42, was produced fused to the NT* solubility tag in BL21*(DE3) *E. coli* cells and the fusion protein NT*- Aβ42 was purified by immobilized metal affinity chromatography (IMAC)^47^. Purified NT*- Aβ42 was cleaved by tobacco etch virus (TEV) protease overnight in cold room, lyophilized and re-dissolved in 7 mol L^−1^ Gdn-HCl. The Aβ42 monomers were isolated in 20 mmol L^−1^ sodium phosphate pH 8.0 with 0.2 mmol L^−1^ EDTA by a Superdex 30 column (GE Healthcare, UK). The monomeric Aβ42 concentration was calculated using an extinction coefficient of 1 424 M^−1^ cm^-1^ for (A_280_−A_300_). For Aβ42 fibrillization, 20 µL solution containing 10 µmol L^−1^ ThT, 3 µmol L^−1^ Aβ42 monomer and different concentrations of the BRICHOS variants at molar ratios 0, 10, 50, and 100% (relative to Aβ42), were added in wells of half-area 384-well microplates with clear bottom (Corning Glass 3766, USA), and incubated at 37 °C under quiescent conditions. The ThT fluorescence was recorded with a 440 nm excitation filter and a 480 nm emission filter using a microplate reader (FLUOStar Galaxy from BMG Labtech, Offenberg, Germany). For all the experiments, aggregation traces were normalized and averaged using four replicates, and data for one set of experiments were recorded from the same plate.

### Aβ42 aggregation kinetics

Aggregation profiles of Aβ42 in the presence of different concentrations of BRICHOS variants were fitted to an empirical sigmoidal equation (Eq. (1))^48,49^, and the aggregation half time ${{\tau }}_{1/2}$ and maximal growth rate ${r}_{\max }$ were extracted.

1 $$F={F}_{0}+{A}/({1+{{\exp }}[{r}_{\max }({\tau }_{\frac{1}{2}}-t)]})$$

where *A* is the amplitude and *F*_*0*_ the base value.

The aggregation traces of the total fibril mass concentration, *M(t)*, is described by the following integrated rate law^32^:

2 $$\frac{{{{{{\boldsymbol{M}}}}}}({{{{{\boldsymbol{t}}}}}})}{{{{{{\boldsymbol{M}}}}}}(\infty )}={{{{{\bf{1}}}}}}-{\left(\frac{{{{{{{\boldsymbol{B}}}}}}}_{+}+{{{{{{\boldsymbol{C}}}}}}}_{+}}{{{{{{{\boldsymbol{B}}}}}}}_{+}+{{{{{{\boldsymbol{C}}}}}}}_{+}\cdot {{{{{\boldsymbol{exp}}}}}} ({{{{{\boldsymbol{\kappa }}}}}}{{{{{\boldsymbol{t}}}}}})}\cdot \frac{{{{{{{\boldsymbol{B}}}}}}}_{-}+{{{{{{\boldsymbol{C}}}}}}}_{+}\cdot {{{{{\boldsymbol{exp}}}}}} ({{{{{\boldsymbol{\kappa }}}}}}{{{{{\boldsymbol{t}}}}}})}{{{{{{{\boldsymbol{B}}}}}}}_{-}+{{{{{{\boldsymbol{C}}}}}}}_{+}}\right)}^{\frac{{{{{{{\boldsymbol{k}}}}}}}_{\infty }^{{{{{\bf{2}}}}}}}{{{{{{\boldsymbol{\kappa }}}}}}{\tilde{{{{{{\boldsymbol{k}}}}}}}}_{\infty }}}\cdot \exp (-{{{{{{\boldsymbol{k}}}}}}}_{\infty }{{{{{\boldsymbol{t}}}}}})$$

where the additional coefficients are functions of λ and κ:

$${C}_{\pm }={\pm \lambda }^{2}/2/{\kappa }^{2}$$

$${k}_{{{\infty }}}=\sqrt{2{\kappa }^{2}/({n}_{2}({n}_{2}+1))+2{\lambda }^{2}/{n}_{C}}$$

$${\widetilde{k}}_{{{\infty }}}=\sqrt{{k}_{{{\infty }}}^{2}{{{{{\rm{\hbox{-}}}}}}}4{C}_{+}{C}_{-}{\kappa }^{2}}$$

$${B}_{\pm }=({k}_{{{\infty }}}\pm {\widetilde{k}}_{{{\infty }}})/2/\kappa$$

which are two combinations of the microscopic rate constants by $\lambda =\sqrt{2\cdot {k}_{+}{k}_{n}\cdot m{\left(0\right)}^{{n}_{C}}}$ and $\kappa =\sqrt{2\cdot {k}_{+}{k}_{2}\cdot m{\left(0\right)}^{{n}_{2}+1}}$. The microscopic rate constants *k*_*n*_, *k*_*+*_, and *k*_*2*_ are the primary nucleation, elongation, and secondary nucleation rate constants, respectively, and the parameters *n*_*C*_ and *n*_*2*_ are the reaction orders for primary and secondary nucleation, respectively.

We identified the microscopic events inhibited by BRICHOS variants by applying Eq. (2) to describe the macroscopic aggregation profiles. The kinetic data at constant Aβ42 concentration with different BRICHOS concentrations were globally analyzed by applying the kinetic nucleation model, where the fit was constrained such that one fitting parameter was held to a constant value across all BRICHOS species/variants concentrations, while the second parameter was the only free parameter. This procedure results in that only one rate constant, i.e. *k*_*n*_, *k*_*+*_ or *k*_*2*_, is the sole fitting parameter^49^.

### Statistics and reproducibility

The protein aggregation data for testing chaperone activities and the ThT fluorescence data are presented as means ± standard deviation, and the aggregation traces are averaged from 3–4 replicates.

### Reporting summary

Further information on research design is available in the Nature Portfolio Reporting Summary linked to this article.

## Supplementary information

Supplementary Information

Description of Additional Supplementary Files

Supplementary Data

Reporting Summary

## Data Availability

All data and materials related to this paper are available from the corresponding author on reasonable request. Uncropped gel images are included as Supplementary Fig. 12. The raw data are compiled in Supplementary Data.
