# Supplementary material for: Short hydrophobic loop motifs in BRICHOS domains determine chaperone activity against amorphous protein aggregation but not against amyloid formation
Source: Commun Biol. 2023 May 8;6:497. doi: 10.1038/s42003-023-04883-2 (PMC10167226; doi:10.1038/s42003-023-04883-2)
Supplement: Supplementary file 1 — Supplementary Information [file 42003_2023_4883_MOESM1_ESM.pdf]

Supplementary Information

Short hydrophobic loop motifs in BRICHOS domains determine chaperone activity against amorphous protein aggregation but not against amyloid formation

Gefei Chen[1,#,*], Axel Leppert[1,4,#], Helen Poska[1,2], Harriet E. Nilsson[3,†], Carlos Piedrafita Alvira[1], Xueying Zhong[3], Philip Koeck[3], Caroline Jegerschöld[3], Axel Abelein[1], Hans Hebert[3], Jan Johansson[1,*]

[1]Department of Biosciences and Nutrition, Karolinska Institutet, 141 83 Huddinge, Sweden.

[2]School of Natural Sciences and Health, Tallinn University, Tallinn, Estonia.

[3]School of Engineering Sciences in Chemistry, Biotechnology and Health, Department of Biomedical Engineering and Health Systems, KTH Royal Institute of Technology, 141 52 Huddinge, Sweden.

[4]Present address Department of Microbiology, Tumour and Cell Biology, Karolinska Institutet, 171 65 Solna, Sweden.

[#]These authors contributed equally

[†]Deceased August 29, 2019

[*]Corresponding authors: gefei.chen@ki.se; janne.johansson@ki.se.

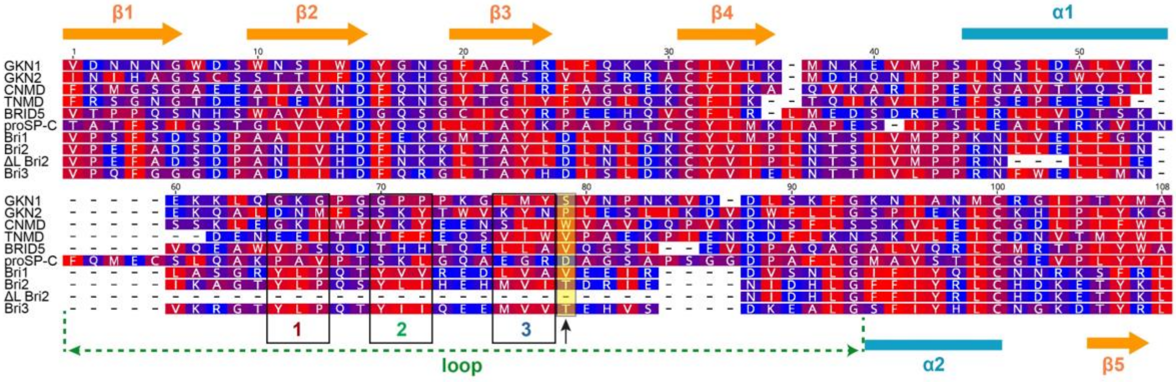

Supplementary Fig. 1 | Amino acid sequence alignment of human BRICHOS domains. Amino acid sequences of human BRICHOS domains aligned using ClustalW embedded in Geneious. Residues are color-coded from blue to red according to increasing hydrophobicity. Gaps have been inserted in order to optimise residue identities. The motifs 1–3 are boxed and color coded and T206 position in Bri2 BRICHOS is labeled with a black arrow and shaded. The secondary structure elements in proSP-C BRICHOS (PDB accession number: 2yad) are shown with arrows and bars. The full loop region is indicated by the dashed green line. The BRICHOS sequences shown are the following: gastrokine 1 (GKN1) (pos. 65–164, GenBank accession no. AAQ89409); GKN2 (pos. 51–151, GenBank accession no. AAQ89027); chondromodulin (CNMD) (pos. 101–201, GenBank accession no. NP_001011705); tenomodulin (TNMD) (pos. 90–186, GenBank accession no. EAX02808); BRICHOS domain containing 5 (BRID5) (pos. 95–193, GenBank accession no. AAT09004); proSP-C (pos. 91–197, GenBank accession no. NP_003009); Bri1 (pos. 130–227, GenBank accession no. BAD97047); Bri2 (pos. 134–231, GenBank accession no. NP_068839); ΔL Bri2 BRICHOS (pos. 134–204, GenBank accession no. XP_013216182). Bri3 (pos. 133–230, GenBank accession no. AAH02424).

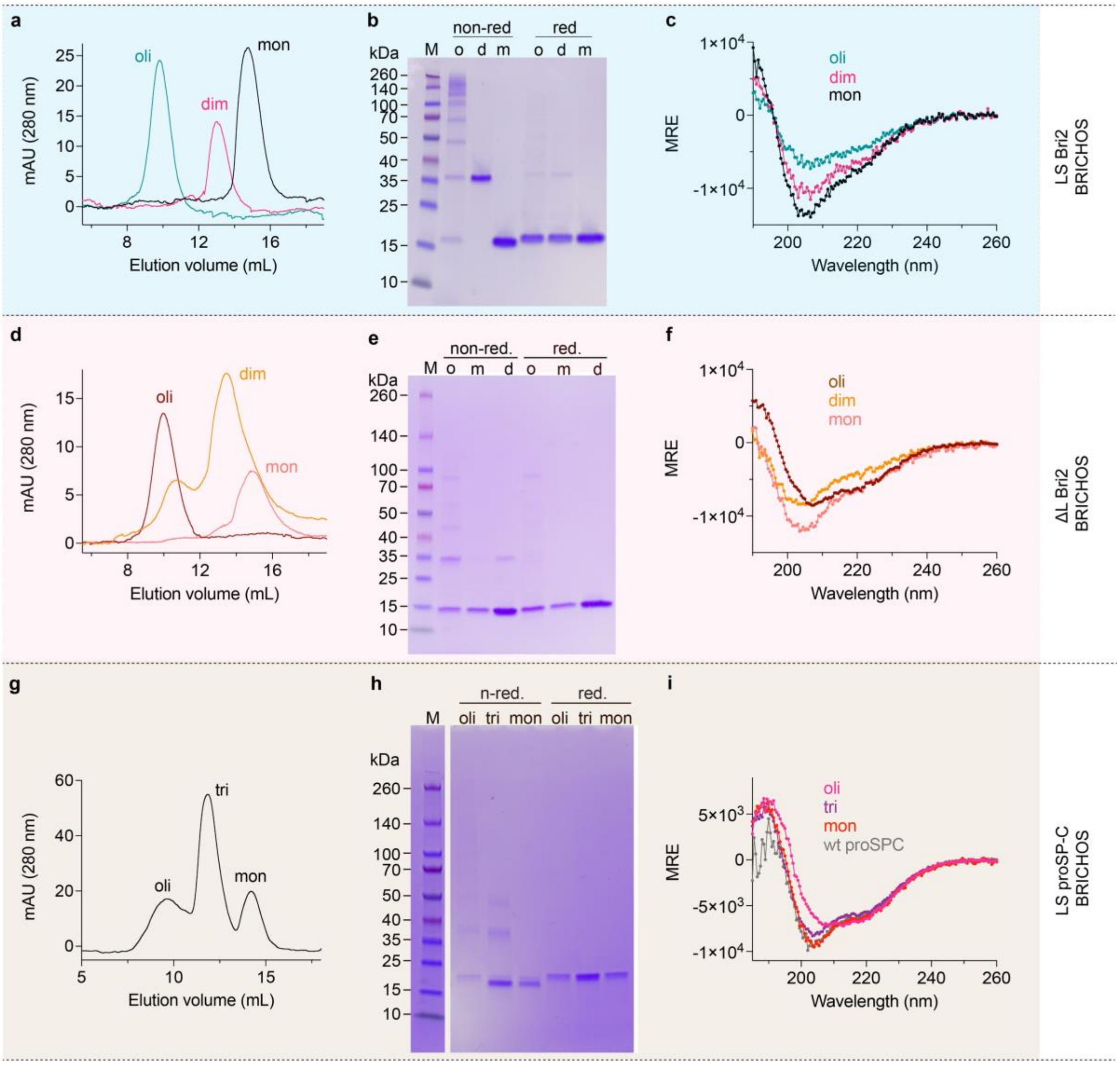

Supplementary Fig. 2 | Properties of BRICHOS loop-swap variants. (**a**) Size exclusion chromatography (SEC), (**b**) SDS-PAGE, (**c**) CD spectra of LS Bri2 BRICHOS oligomer (oli, o), dimer (dim, d) and monomer (mon, m). (**d**) SEC, (**e**) SDS-PAGE, (**f**) CD spectra of ΔL Bri2 BRICHOS oligomer (oli, o), dimer (dim, d) and monomer (mon, m). (**g**) SEC, (**h**) SDS-PAGE (**i**) CD spectra of LS proSP-C BRICHOS oligomer (oli), trimer (tri) and monomer (mon) and wt proSP-C BRICHOS. M: protein ladder.

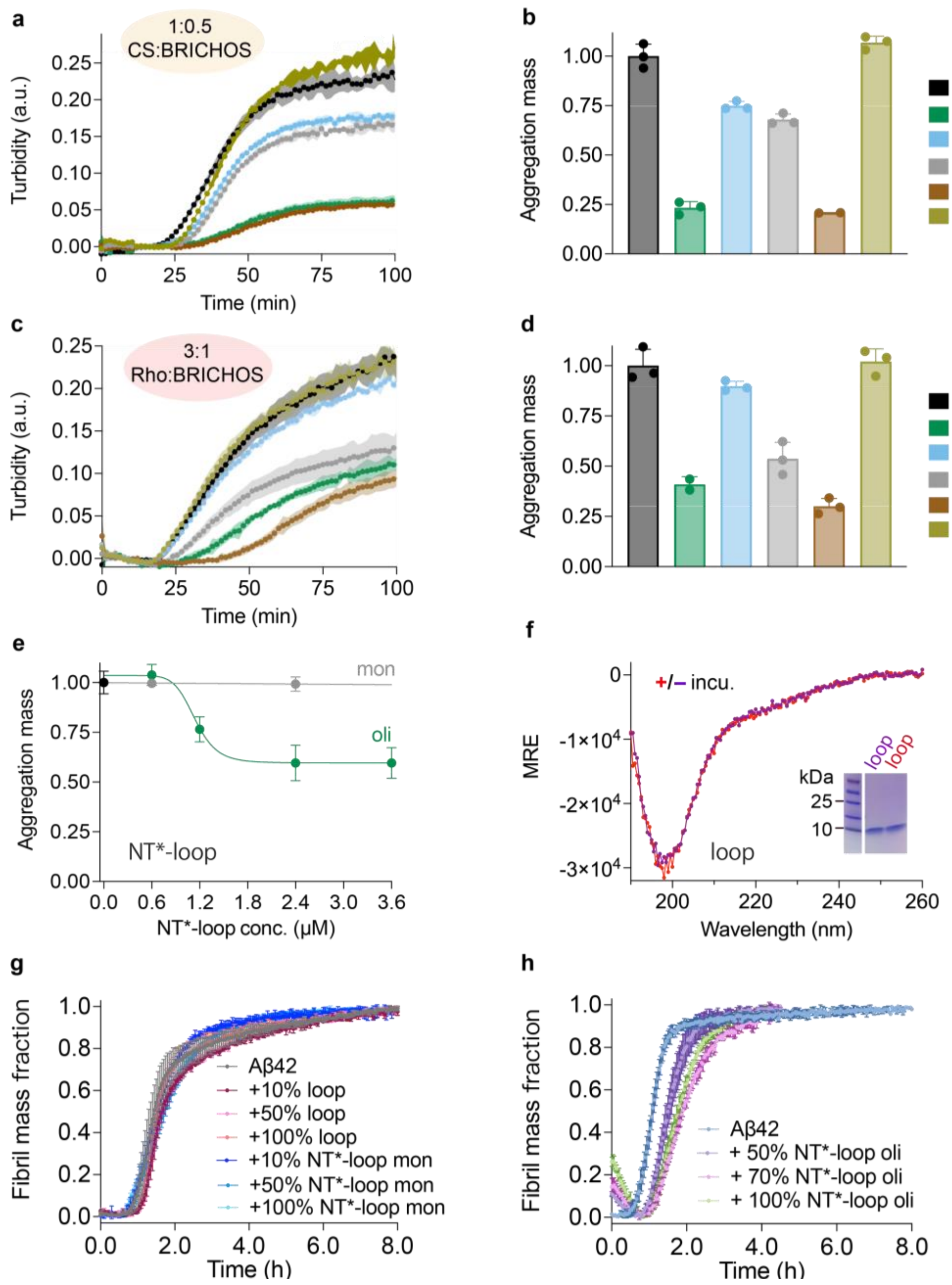

Supplementary Fig. 3 | Ability of BRICHOS loop variants to suppress amorphous protein aggregation. (**a**) Aggregation traces of 1.2 μmol L^{-1} CS at 45°C alone and with the different loop variants at a molar ratio of 1:0.5 (CS: BRICHOS), color-coded as in (**b**). (**b**) Aggregation mass determined from the areas under curves in (**a**). (**c**) Aggregation traces of 3 μmol L^{-1} Rho at 45°C alone and with the different loop variants at a molar ratio of 3:1 (Rho: BRICHOS), color-coded as in (**d**). (**d**) Aggregation mass determined from the areas under curves in (**c**). (**e**) Aggregation mass of 1.2 μmol L^{-1} CS at 45°C in the presence or absence of NT*-loop fusion

protein monomer (mon, linear fit) and oligomer (oli, sigmoidal fit). (**f**) CD spectra of the isolated loop peptide before and after overnight incubation at 37°C. The inset shows the SDS-PAGE analysis of the loop peptide with (right lane) and without (left lane) incubation. (**g and h**) Aggregation kinetics of 3 μmol L^{-1} Aβ42 monomers monitored by ThT fluorescence in the absence or presence of different molar ratios of recombinant loop peptide, or NT*-loop fusion protein monomer or oligomers. The data are presented as means ± standard deviations.

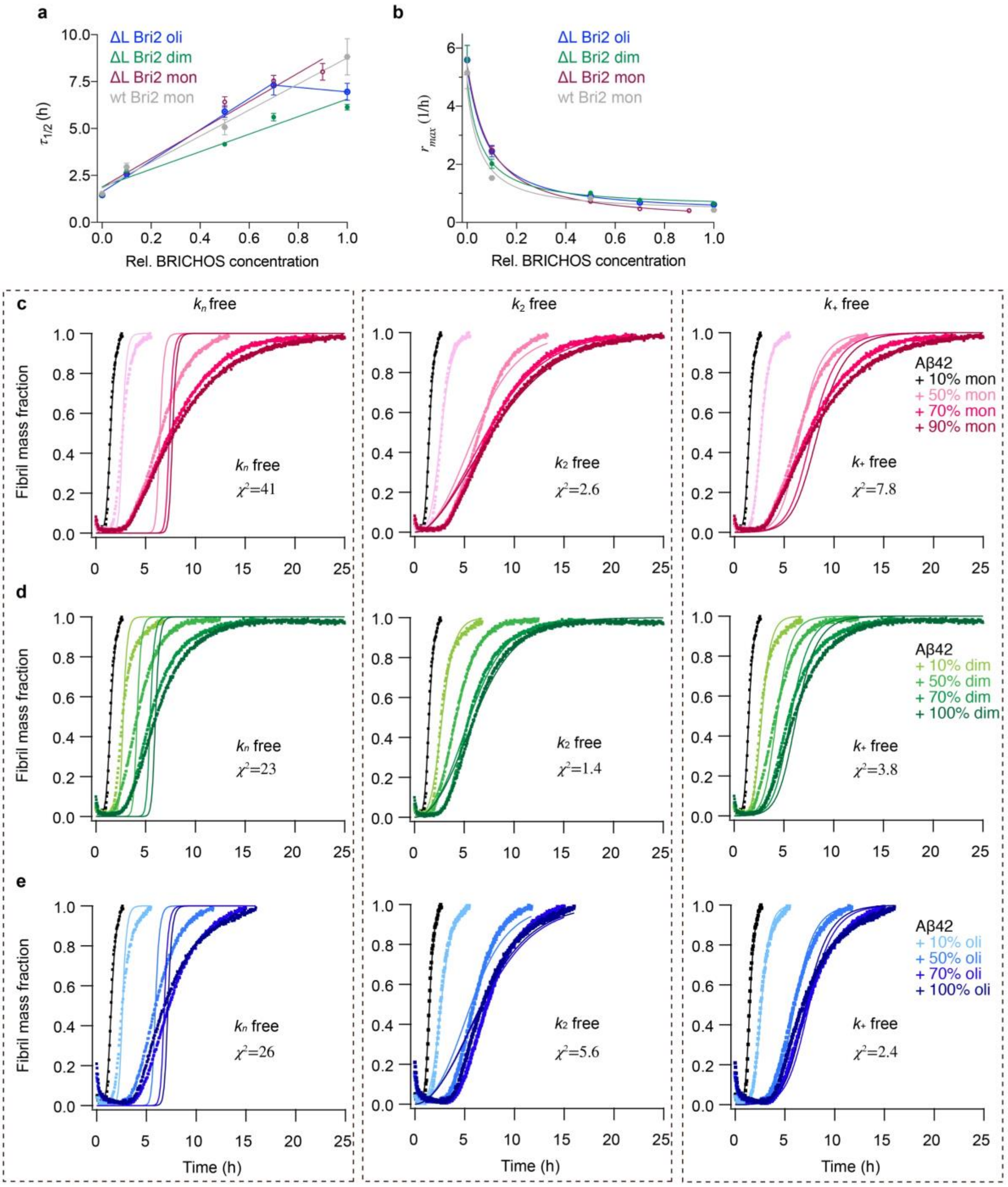

Supplementary Fig. 4 | ΔL Bri2 BRICHOS ability to inhibit Aβ42 fibril formation. (**a**) Values for $\tau_{1/2}$ and (**b**) r_{max} extracted from the sigmoidal fits of Aβ42 aggregation traces in the presence of different concentrations of ΔL Bri2 BRICHOS oligomer (oli), dimer (dim), monomer (mon) and wt Bri2 BRICHOS monomer monitored by ThT fluorescence. **(c–e)** Aggregation kinetics of 3 µmol L^{-1} Aβ42 in the presence of ΔL Bri2 BRICHOS monomer (mon, **c, red**), dimer (dim, **d, green**) and oligomer (oli, **e, blue**) at different molar ratios. The

global fits (solid lines) of the aggregation traces (squares) were constrained such that only one rate constant is the free fitting parameter, indicated in each panel. χ^2 values describe the quality of the fits.

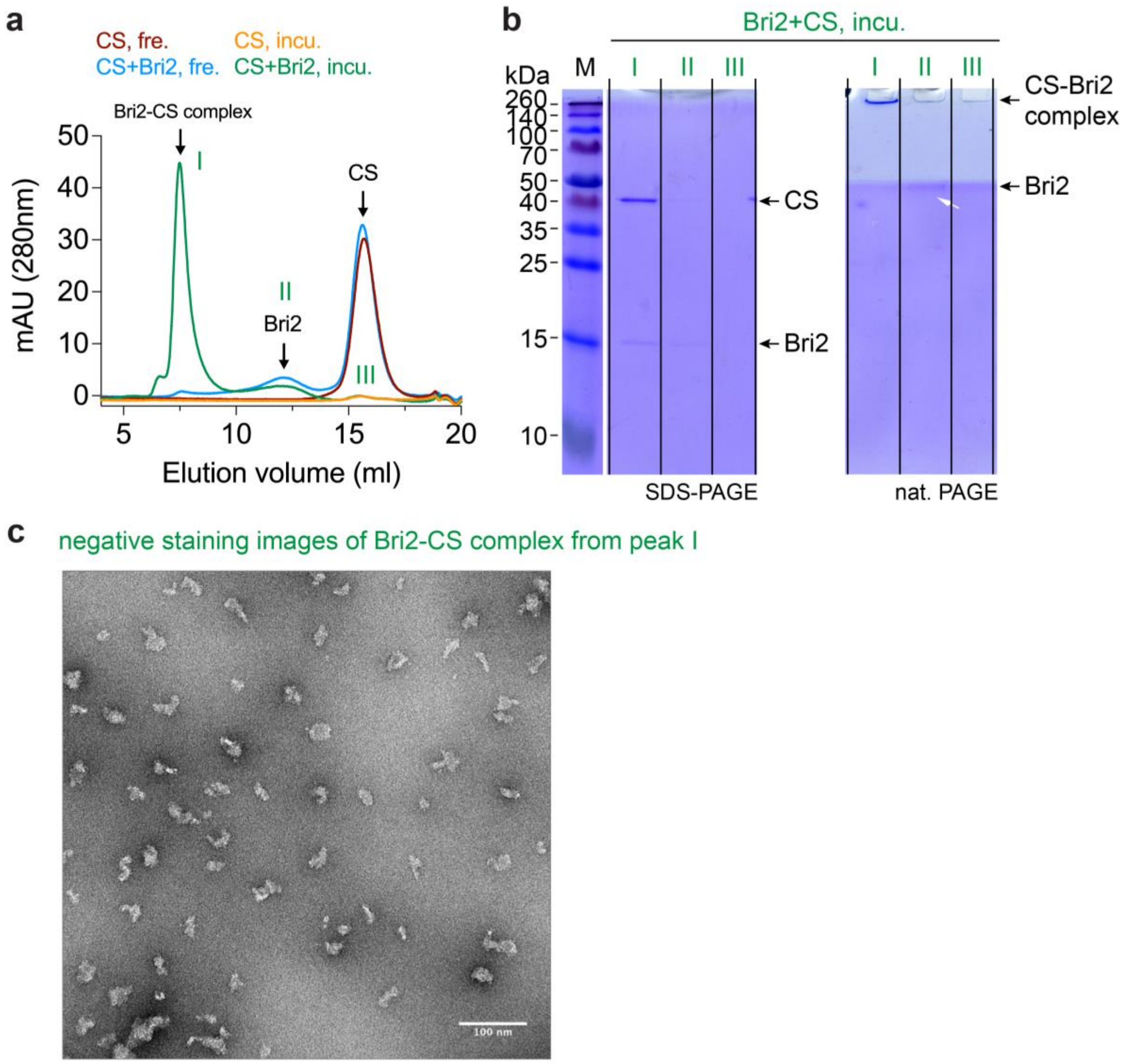

Supplementary Fig. 5 | Rh Bri2 BRICHOS oligomer and monomer interactions with thermo-unfolded CS and bis-ANS. (**a**) SEC of fresh CS with and wihout rh Bri2 BRICHOS oligomer (Bri2), and of CS incubated with and without rh Bri2 BRICHOS oligomer. I, II, and III in green indicate fractions collected for CS incubated with rh Bri2 BRICHOS oligomer. (**b**) SDS-PAGE and native PAGE of SEC-isolated CS incuabted with rh Bri2 BRICHOS oligomer

from (**a**). M for protein marker. (**c**) Negative-staining EM micrograph of rh Bri2 BRICHOS oligomer-CS complex present in fraction I in (**a**).

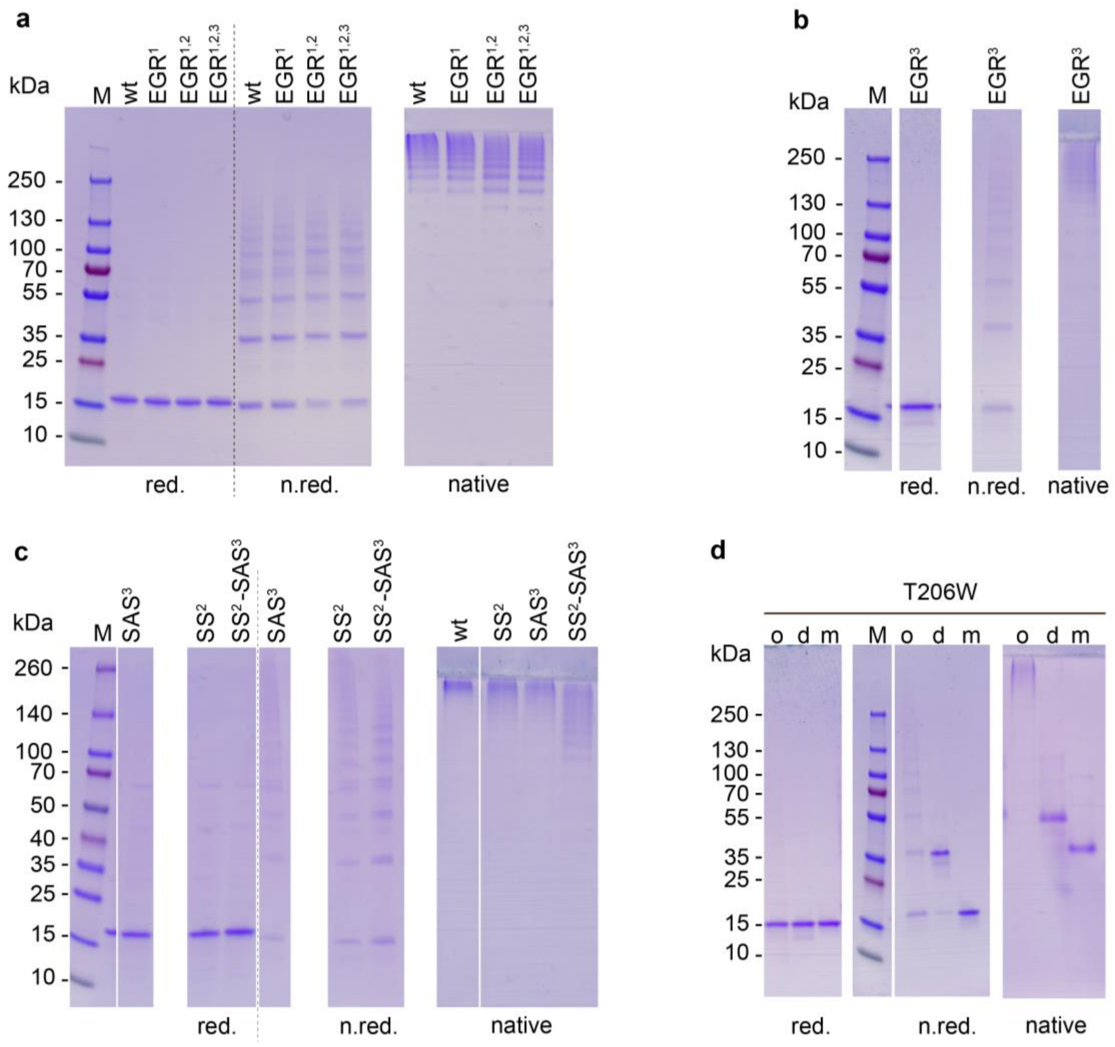

Supplementary Fig. 6 | Characterisation of human Bri2 BRICHOS mutants. (a–d) SDS and native PAGE of oligomers of (**a**) Bri2 BRICHOS EGR^1, $EGR^{1,2}$ and $EGR^{1,2,3}$, (**b**) EGR^3, (**c**) SS^2, SAS^3 and SS^2-SAS^{33}, and (**d**) oligomer (o), dimers (d), and monomers (m) of T206W analyzed under reducing (red.) and non-reducing (n.red.) conditions M: protein ladder.

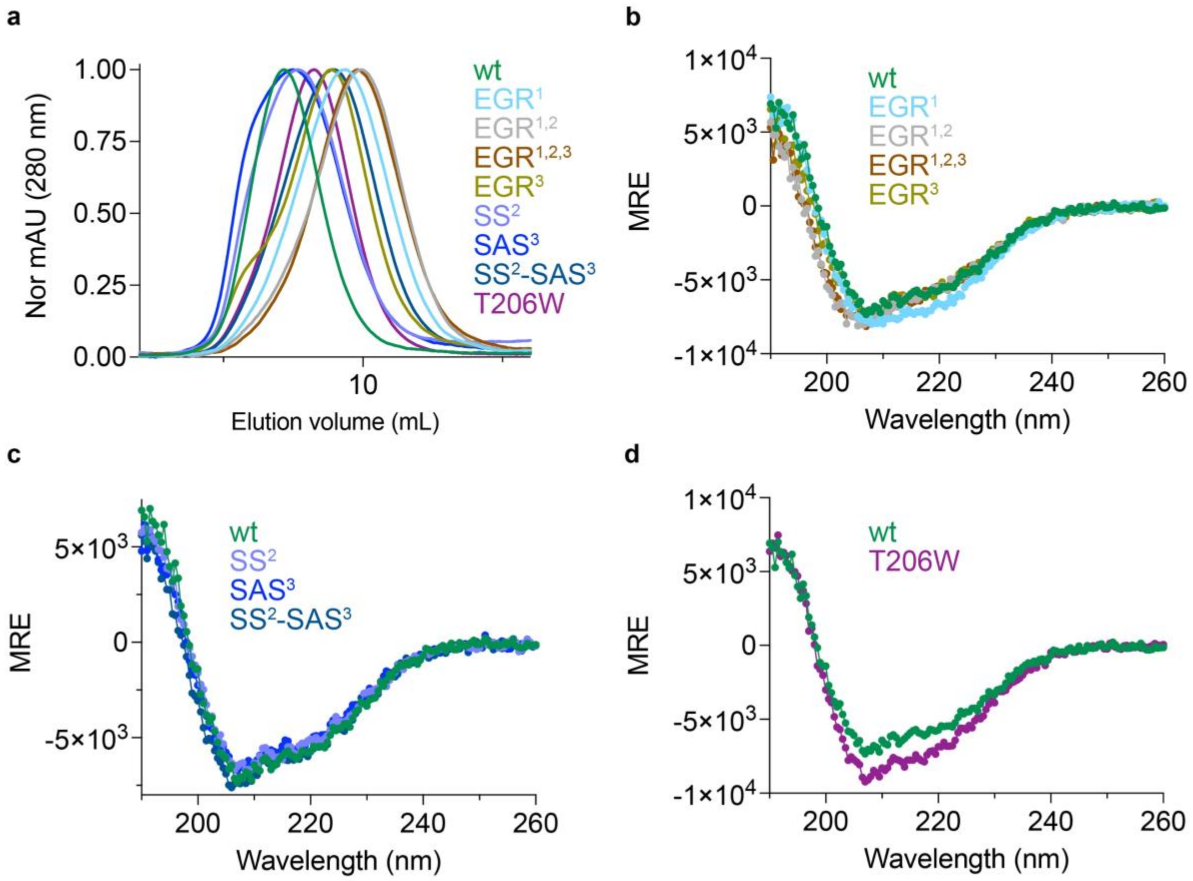

Supplementary Fig. 7 | SEC and CD measurements of human Bri2 BRICHOS mutants. (**a**) SEC analysis of oligomers of wt Bri2 BRICHOS and corresponding mutants. (**b**) CD spectra of wt Bri2 BRICHOS and indicated mutants. (**c**) CD spectra of wt Bri2 BRICHOS and indicated mutants. (**d**) CD spectra of wt Bri2 BRICHOS and T206W mutant.

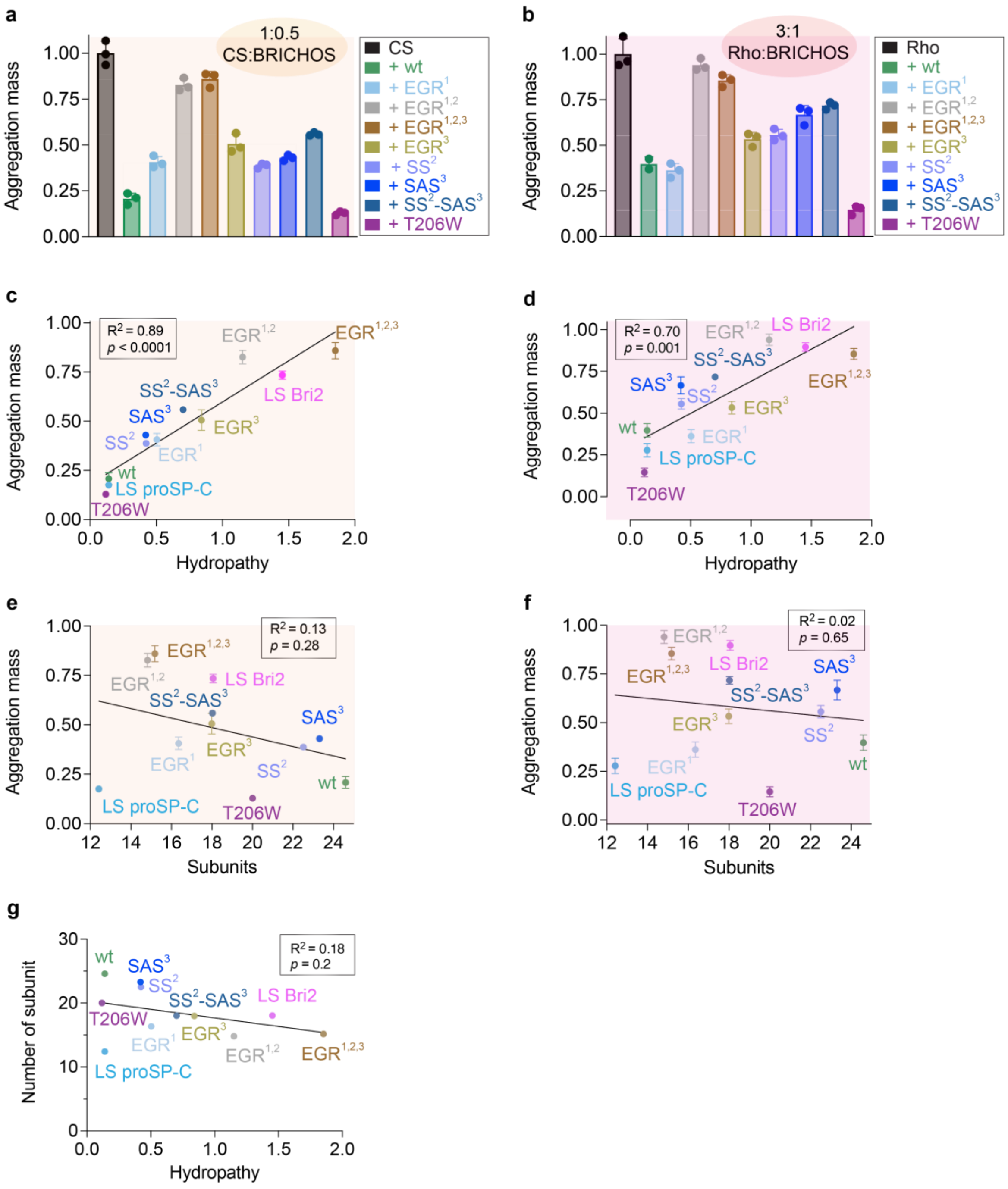

Supplementary Fig. 8 | Ability of BRICHOS mutants to suppress amorphous protein aggregation. (**a**) Aggregation mass of 1.2 µmol L^{-1} CS at 45°C in the presence or absence of the indicated loop mutants at 1:0.5 (CS: BRICHOS) molar ratio. (**b**) Aggregation mass of 3 µmol L^{-1} Rho at 45°C with and without the indicated loop mutants at a molar ratio of 3:1 (Rho: BRICHOS). (**c**) Correlation analysis between the CS aggregation mass (data from **a**) and hydropathy for each BRICHOS mutant. (**d**) Correlation analysis between the Rho aggregation

mass (data from **b**) and hydropathy for each BRICHOS mutant. (**e**) Correlation analysis of the CS aggregation mass (data from **a**) and the number of constituent subunits for each BRICHOS mutant (data from **Supplementary Fig. 7a**). (**f**) Correlation analysis of the Rho aggregation mass (data from **b**) and the number of constituent subunits for each BRICHOS mutant (data from **Supplementary Fig. 7a**). (**g**) Correlation analysis of the number of constituent subunits for each BRICHOS mutant (data from **Supplementary Fig. 7a**) and hydropathy for each BRICHOS mutant. The data are presented as means ± standard deviations. For hydropathy values (**c–d and g**), combined motifs 1–3 plus T206 or W206 for each specific BRICHOS mutant were considered, respectively. Panels with data for the same substrate/BRICHOS ratio have the same color.

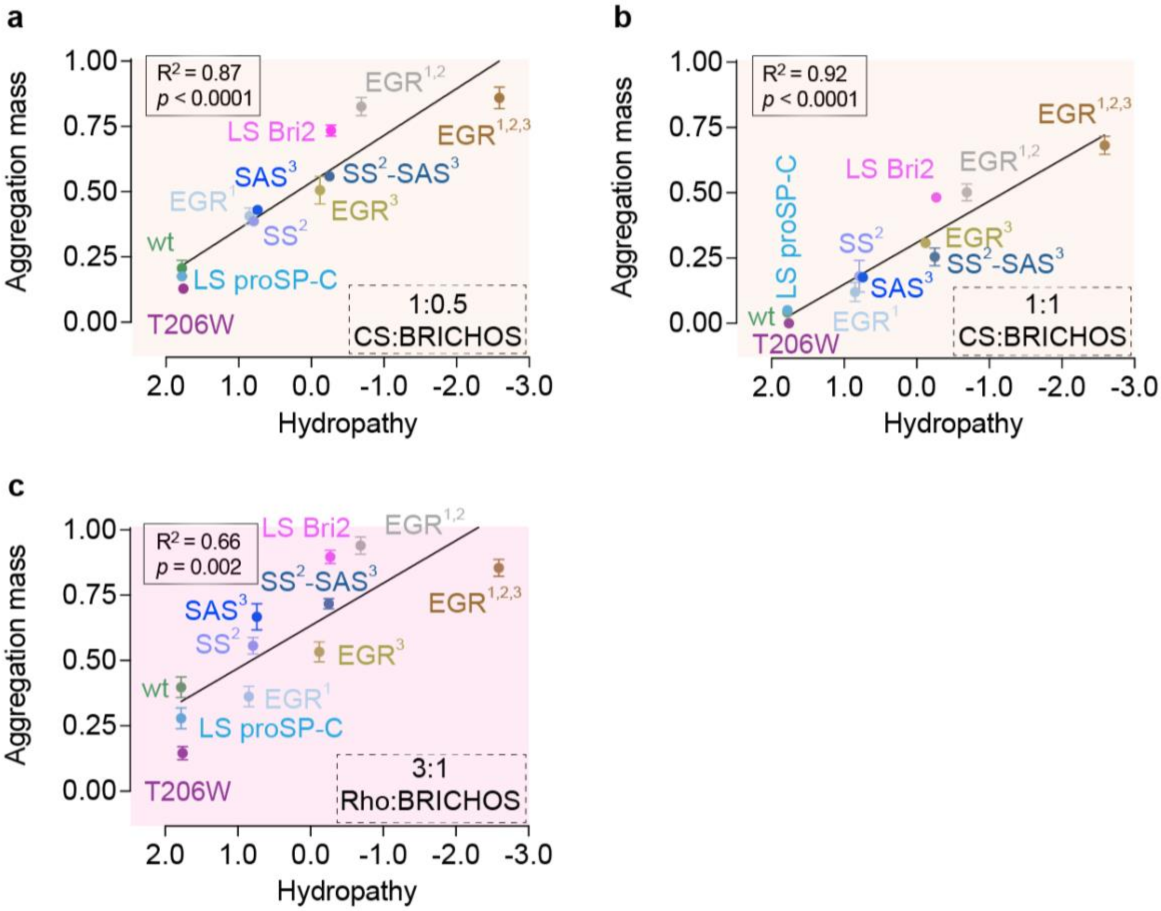

Supplementary Fig. 9 | Correlation analysis of BRICHOS loop hydropathy and ability to suppress amorphous protein aggregation. Correlation analysis between the CS aggregation mass at (**a**) CS:BRICHOS=1:0.5 (data from **Supplementary Fig. 8a**), (**b**) CS:BRICHOS=1:1 (data from **Fig. 3c**), or (**c**) Rho aggregation mass at Rho:BRICHOS=3:1 (data from **Supplementary Fig. 8b**) and hydropathy calculated using the Kyte-Doolittle scale[1] for motifs 1-3 and Thr206 or Trp206 for each BRICHOS loop-swap variant or mutant.

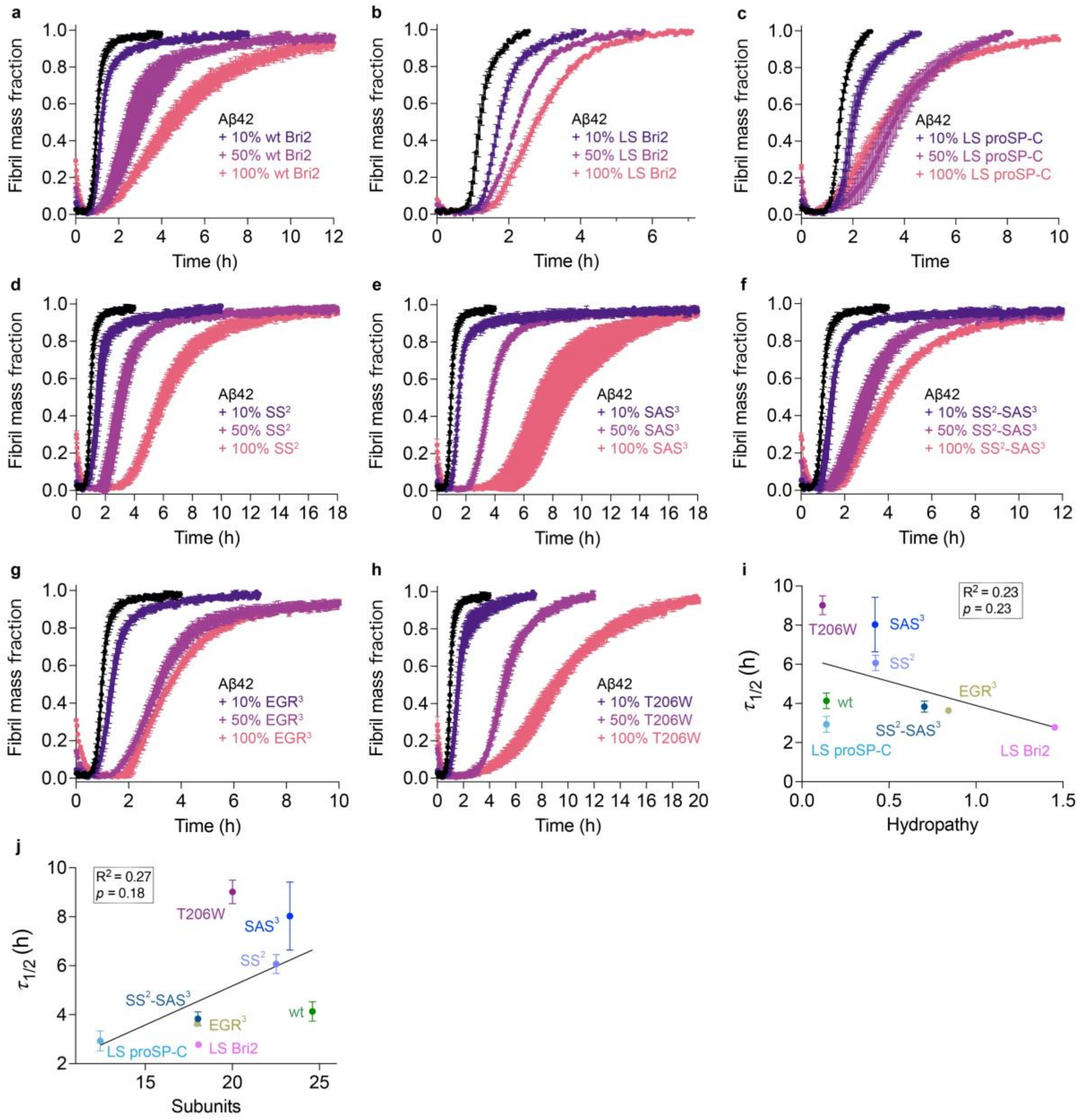

Supplementary Fig. 10 | Anti-amyloid fibril formation activity of the Bri2 BRICHOS mutants. Aggregation kinetics of 3 µmol L^{-1} Aβ42 monomers in the absence or presence of oligomers of (**a**) wt Bri2 BRICHOS, (**b**) LS Bri2 BRICHOS, (**c**) LS proSP-C BRICHOS, (**d**) SS^2, (**e**) SAS^3, (**f**) SS^2-SAS^3, (**g**) EGR^3, and (**h**) T206W mutants at 10%, 50% and 100% molar ratios monitored by ThT fluorescence. (**i,j**) Correlation between $\tau_{1/2}$ extracted by sigmoidal fitting from traces at 100% molar ratio in (**a–h)** and (**i**) hydropathy of the combined motifs 1–3 plus T206 or W206 for each variant, and (**j**) the number of constituent subunits for each

BRICHOS mutant (data from **Supplementary Fig. 7a**). The data are presented as means ± standard deviations.

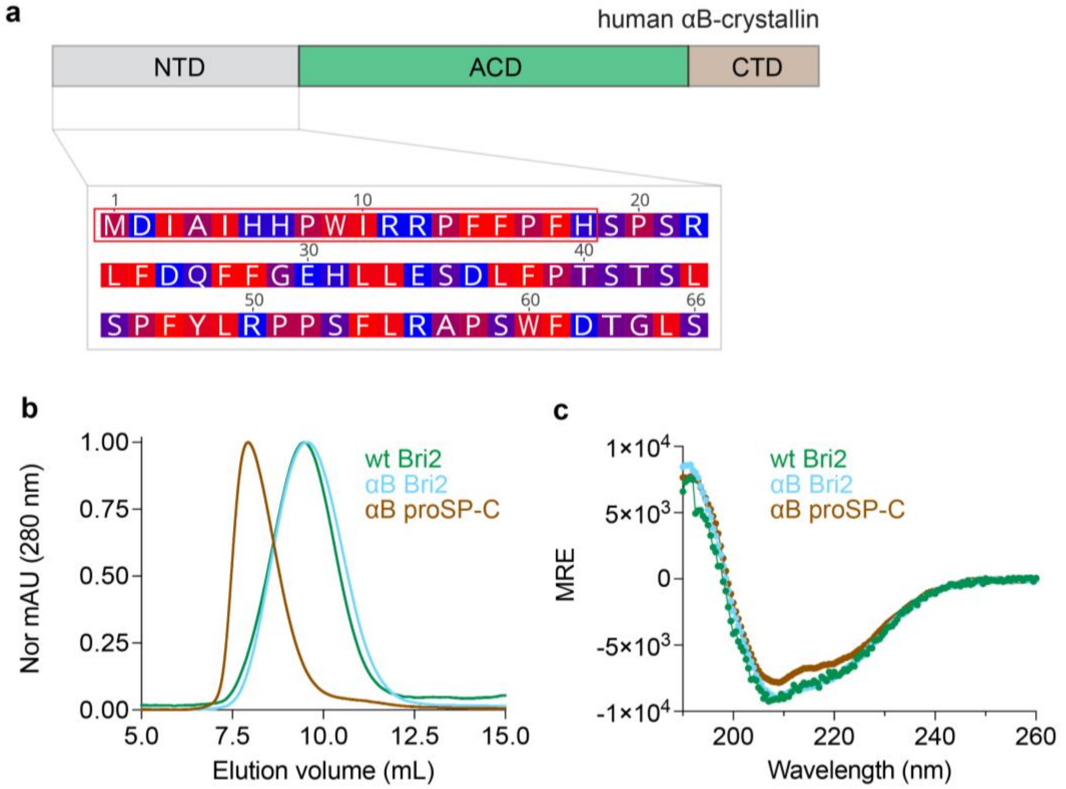

Supplementary Fig. 11 | Properties of αB BRICHOS chimeras. (**a**) Architecture of human αB-crystallin (NCBI accession number: P02511). The N-terminal domain (NTD, amino acid residues 1–66) is color-coded from blue to red according to increasing hydrophobicity. The N-terminal 18 residues that replaced the core loop regions in αB-Bri2 or αB-proSP-C BRICHOS chimeras are boxed. ACD, α crystallin domain. CTD, C-terminal domain. (**b**) SEC analysis of oligomers of wt Bri2, αB Bri2 and αB proSP-C BRICHOS chimeras. (**b**) CD spectra of oligomers of wt Bri2, αB Bri2 and αB proSP-C BRICHOS chimeras.

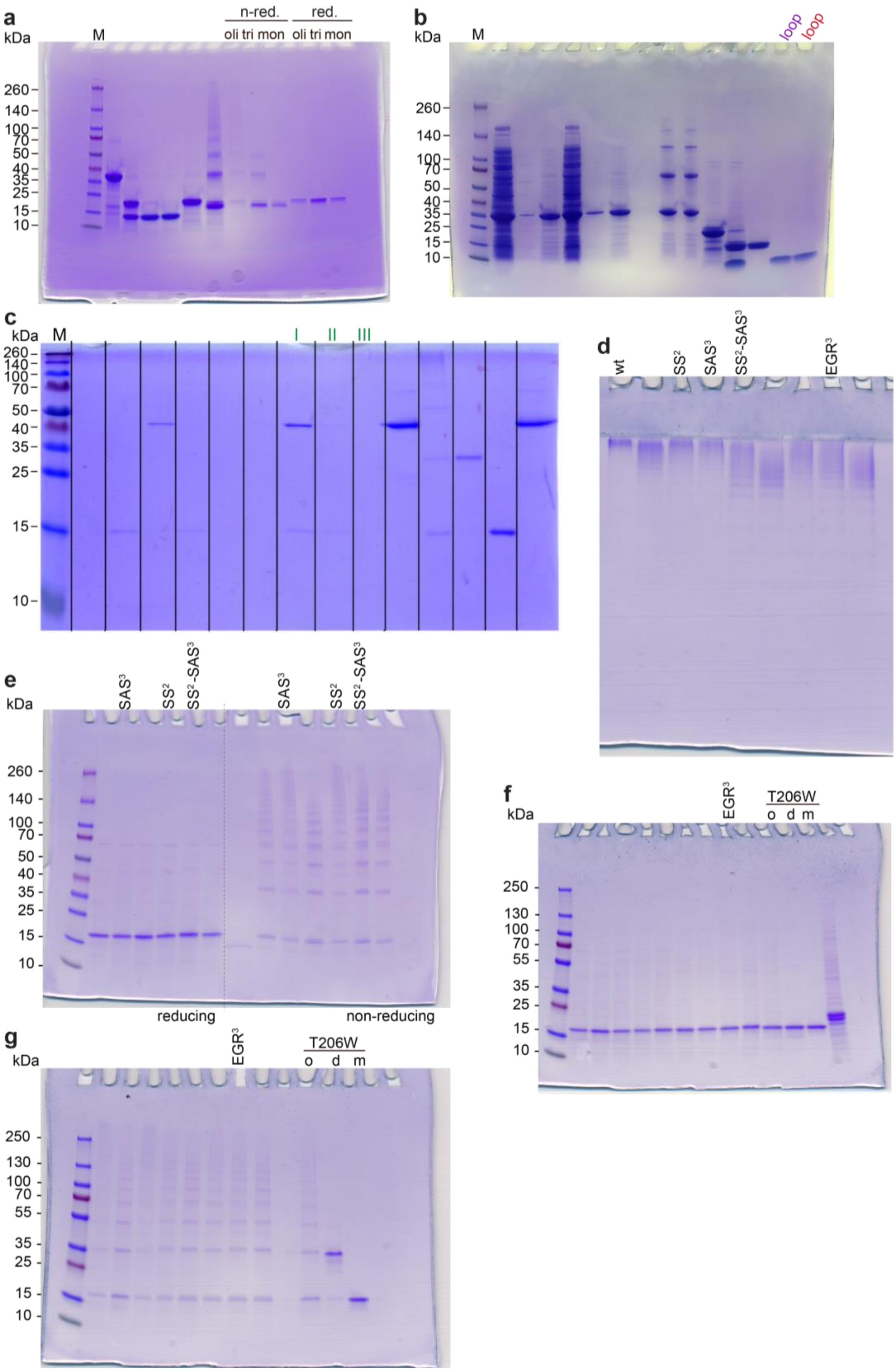

Supplementary Fig. 12 | Uncropped PAGE gels. (**a**) SDS-PAGE of **Supplementary Figure**

2h. (**b**) SDS-PAGE of **Supplementary Figure 3f**. (**c**) SDS-PAGE of **Supplementary Figure 5b**. (**d**) Native PAGE of **Supplementary Figure 6b** and **c**. (**e**) SDS-PAGE of **Supplementary Figure 6c**. (**f**) SDS-PAGE of Supplementary **Figure 6b** and **d**. (**g**) SDS-PAGE of **Supplementary Figure 6b** and **d**.

Supplementary Reference

1 Kyte, J. & Doolittle, R. F. A simple method for displaying the hydropathic character of a protein. *J. Mol. Biol.* **157**, 105-132 (1982).
