# Supplementary material for: Short hydrophobic loop motifs in BRICHOS domains determine chaperone activity against amorphous protein aggregation but not against amyloid formation
Source: Commun Biol. 2023 May 8;6:497. doi: 10.1038/s42003-023-04883-2 (PMC10167226; doi:10.1038/s42003-023-04883-2)
Supplement: Supplementary file 2 — Description of Additional Supplementary Files [file 42003_2023_4883_MOESM2_ESM.pdf]

Description of Additional Supplementary Files

File name: Supplementary Data

Description: Raw data for figures 2b and 3c.
